# Study on the correlations of different clinical types with imaging findings at initial diagnosis and clinical laboratory indexes in COVID-19 patients

**DOI:** 10.12669/pjms.38.6.5091

**Published:** 2022

**Authors:** Hongwei Ren, Xiaobo Zhang, Yu Tang, Tao Yan, Yuan Liu

**Affiliations:** 1 Hongwei Ren, Department of Radiology, Fifth Medical Center of PLA General Hospital, Beijing 100039, China; 2 Xiaobo Zhang, Department of Radiology, The First Medical Center of Chinese PLA General Hospital, Chinese PLA Medical School, Beijing 100853, China; 3 Yu Tang, Department of Ultrasound, Beijing Tiantan Hospital, Capital Medical University, Beijing 100070, China; 4 Tao Yan, Fifth Medical Center of PLA General Hospital, Beijing 100039, China Department of International Liver Disease,; 5 Yuan Liu, Department of Radiology, Chinese PLA Medical School, Beijing 100853, China. Fifth Medical Center of PLA General Hospital, Beijing 100039, China

**Keywords:** Coronavirus Disease 2019, Computed tomography, Imaging features, Artificial intelligence, Clinical characteristics

## Abstract

**Objectives:**

To investigate the correlations of initial lab and imaging findings in COVID-19 patients of different clinical types.

**Methods:**

We retrospective analyzed patients confirmed with COVID-19 in the Fifth Medical Center of the People’s Liberation Army (PLA) General Hospital between February to April 2020, selected a total of 58 (N) patients with lab and imaging examinations that met the study criteria, using Artificial intelligence (AI) software to calculate the percentage of COVID-19 lesions in the volume of the whole lung, then the correlations of general information, initial chest CT examination after admission and laboratory examinations were analyzed.

**Results:**

The 58 (N) COVID-19 patients were divided into mild group [41(n) cases]: and severe group [17(n) cases]: according to patient’s condition. CT findings of the severe group and mild group mainly included single or multiple ground glass opacity (GGO), with lesions mainly distributed in the periphery of lungs or GGO mixed with consolidation, with lesions involved in peripheral and central areas of both lungs, accompanied other signs. A significant difference in CRP, IL-6, D-D, GGT was observed between the two groups (p < 0.05). The ratios regarding lymphocyte abnormality and neutrophil abnormality in the severe group were higher than those in the mild group (p < 0.05).

**Conclusion:**

The CT features at initial diagnosis of COVID-19 were mainly characterized by multiple GGO with or without partial consolidation in both lungs, with the lesions mainly distributed at the subpleural regions. Some lab test indexes were correlated with the clinical types of COVID-19.

## INTRODUCTION

A Novel coronavirus of 2019 (nCoV-2019) (β-genus) was found to be mainly transmitted through droplets and contacts, and 2019-nCoV could cause respiratory tract, intestinal tract, liver and nervous system diseases, posing a serious threat to global public health security.^1^-^4^

According to the Guidelines on the Novel Coronavirus-Infected Pneumonia Diagnosis and Treatment (trial version 7th), the COVID-19 was clinically classified into mild type, common type, severe type and critical type.^5^

The objective of this study was to improve the understanding of COVID-19 and provide important reference value for clinical early diagnosis and severity assessment of COVID-19 patients by analyzing the clinical features and imaging data of patients with mild COVID-19 (mild type and common type) and severe COVID-19 (severe type and critical type).

## METHODS

We performed a retrospective study for patients with COVID-19 admitted to the Fifth Medical Center of the PLA General Hospital. In patient analyses, the patients with COVID-19 during the period of February 2020 – April 2020 were included.

### Inclusion criteria:

• Patient age ≥ 18 years;
• In accordance with the diagnostic criteria and clinical classification criteria of the Guidelines on the Novel Coronavirus-Infected Pneumonia Diagnosis and Treatment (trial version 7th);^5^
• With initial lab examinations of routine, biochemistry and cardiac function indexes after admission
• With initial CT examination of chest after admission.

### Exclusion criteria:

• Patient age < 18 years;
• Lack of initial lab examinations enrolled in inclusion criteria;
• The CT images had artifacts or poor image quality impact assessment.

Fifty eight patients were enrolled in this study. They were divided into mild group (mild type and common type) and severe group (severe type and critical type) according to patient’s condition.

LightSpeed VCT64 scanner (GE Medical systems, USA) was used for CT examination. Patients were scanned in supine position. The scanning range was from the apex of lung to bilateral costophrenic angles. Scanning parameters: tube voltage 120 kV, auto-milliampere technique (40-250 mA), noise index (NI) = 25, pitch 0.984: 1, matrix 512 × 512 and slice thickness 5 mm. Lung window setting: window width/level: 2000/-600 HU, mediastinal window 350/40 HU, axial reconstruction of lung window, slice thickness 0.625 mm.

CT images were estimated by two experienced radiologists independently. In case of disagreement, a consensus was finally reached by consultation. CT findings of COVID-19 patients were mainly described according to the following characteristics:

• Distribution of lesions
• Llocation of lesions;
• Other common signs: air bronchogram sign, vascular thickening sign, crazy-paving sign, etc.;
• Extrapulmonary manifestations: pleural effusion or not; and

The percentage of pneumonia lesions in the volume of the whole lung quantitatively calculated with the use of Biomind COVID-19 edition AI software (Beijing Andeyizhi Science & Technology Co., Ltd.).

Venous blood samples were collected from all patients in the early morning under the fasting stage. Routine blood test, interleukin-6 (IL-6), biochemical indexes and cardiac function indexes were detected from peripheral blood samples. Blood cell analyzer, electrochemiluminescence immunoassay analyzer and automatic biochemical analyzer were used.

All data were analyzed by the SPSS 21.0 statistical software. The qualitative data were expressed by frequency and rate, and chi-square test or Fisher exact probability method were applied to the comparison between groups. The quantitative data were expressed by *x̅± s* for normal distribution and median (quartile range) [M (Q)] for non-normal distribution, and the comparison between groups was carried out by independent sample t test and Wilcoxon rank sum test, respectively. *P* < 0.05 indicted that the difference was statistically significant.

The research scheme was submitted to the Institutional Ethics Committee of Fifth Medical Center of Chinese PLA General Hospital, 11 experts discussed in meeting and approved it (Dated: July 24, 2021). All participants were contacted to inform them of the content and purpose of the study, after that they signed informed consents.

## RESULTS

The clinical features of patients included in the study: there were 30 (n) males and 28 females aged 15-85 years old, with an average age of 49.1 ± 16.9 years old. There were 41 (n) cases with mild COVID-19 and 17(n) cases with severe COVID-19. In addition, there were 16 (n) cases with underlying diseases (27.6%), including 3 (n) cases with cardiovascular diseases (5.2%), 7(n) cases with diabetes mellitus (12.1%), 12(n) cases with hypertension (20.7%) and 3(n) cases with diabetes mellitus associated with hypertension (5.2%). The main clinical symptoms were manifested as fever, cough, fatigue, etc.

Of the 41(n) patients in the mild group, there were 31(n) patients (75.7%) with lesions mainly involved in the both lungs (Fig.1 A-C). The CT manifestations of patients with severe/critical COVID-19 (Fig.1 D-F) were multiple, patchy and mixed density shadow, with lesions often involving the peripheral and central regions of both lungs. The percentage of pneumonia lesions in the volume of the whole lung was significantly lower in patients with mild COVID-19 than that in patients with severe COVID-19 (*p* < 0.05). The CT features of COVID-19 patients with different clinical types were shown in Table-I. There were significant differences in ANC, ALC, CRP, IL-6 and PCT between the two groups (*p* < 0.05) (Table-II).

**Fig.1A:**
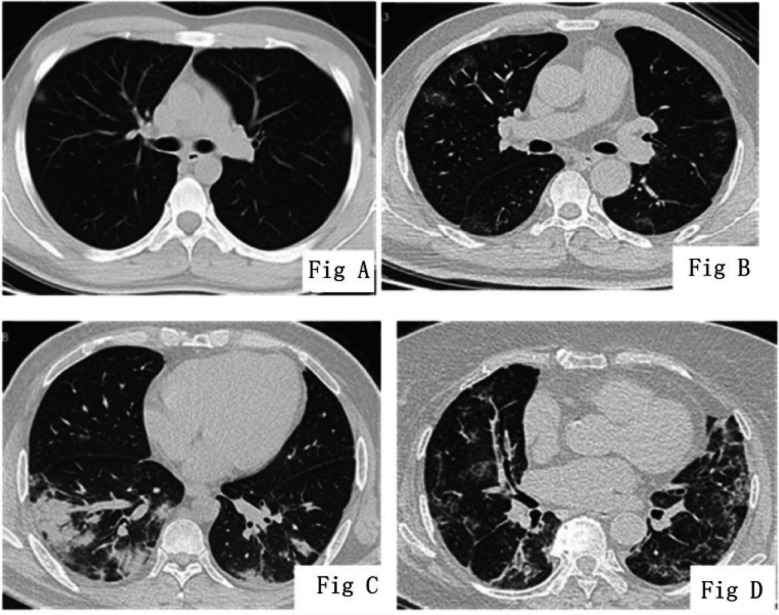
COVID-19 (common type), male, 27 years old, with the visibility of ground-glass nodular (GGN) shadow at the subpleural regions of the upper lobes of both lungs, with h alo sign-like change around. Fig.1B: COVID-19 (common type), male, 59 years old, with the visibility of multiple ground-glass opacity (GGO) at the bilateral subpleural regions and fuzzy boundary, as well as thickened vascular shadows in some lesions. Fig.1C: COVID-19 (common type), female, 44 years old, with the presence of patchy consolidation shadow in the lower lobes of both lungs, air bronchogram sign in the lesions and halo sign-like change around the lesions at the lower lobe of the right lung. Fig.1D: COVID-19 (severe type), female, 74 years old, with the visibility of multiple ground-glass opacity (GGO) in both lungs with partial consolidation and interlobular septal t hickening, showing a grid-like change.

**Fig.1E:**
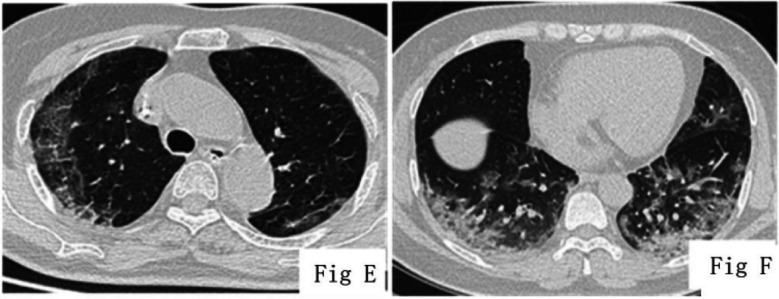
COVID-19 (critical type), male, 79 years old, with the visibility of band-like high-density shadows at the subpleural regions of the upper lobes of both lungs, parallel to the pleura, and with the presence of interlobular septal thickening in the lesions, showing a crazy-paving sign-like change. Fig.1F: COVID-19 (critical type), female, 50 years old, with the visibility of multiple GGO and consolidation in both lungs, as well as air bronchogram sign in the lesions.

**Table-I:** Comparisons of chest CT images between the mild group and the severe group (n).

Variables	Mild group (common type) (n=34)	Severe group (n=17)	Test value	P-value
Distribution of lesions, n				0.542
Left lung only	0	0		
Right lung only	3	0		
Botd lungs	31	17		
Density of lesions				0.831
GGO	23	12	0.04	
Consolidation	8	13	13.114	
Mixed GGO	17	14	4.977	
Pleural effusion	2	5	-	0.034
Percentage of pneumonia lesions in tde volume of tde whole lung (%)	2.42(0.04~4.19)	11.54 (2.26~18.64)	10.662	0.001

***Note***: COVID-19, Corona Virus Disease 2019; GGO, ground-glass opacity; -, the Fisher exact probability method was used, without statistics.

**Table-II:** Comparisons in laboratory indexes in COVID-19 patients between the mild group and the severe group.

Grouping	Mild group	Severe group	Test value	P-value
Blood routine test and inflammatory factors				
WBC (×10^9^/L)	4.95 (4.19~6.42)	6.76 (3.65~9.46)	-1.74	0.082
< 4	10/41 (24.4%)	5/17 (29.4%)	-	0.747
4-10	29/41 (70.7%)	10/17 (58.8%)	0.774	0.379
> 10	2/41 (4.8%)	2/17 (11.8%)	-	0.055
ANC (×10^9^/L)	2.76 (2.14~4.11)	5.52 (2.78~8.31)	-2.673	0.008
> 7	2/41 (4.8%)	4/17 (23.5%)	-	0.002
ALC (×10^9^/L)	1.59 ± 0.68	0.79 ± 0.41	4.641	0.000
< 0.8	5/41 (12.2%)	9/17 (52.9%)	-	0.002
CRP (mg/l)	2.35 (1.18~7.17)	8.26 (3.33~8.26)	-2.787	0.005
> 8.2	10/41 (24.4%)	8/17 (47.1%)	2.889	0.085
IL-6 (pg/ml)	5.18 (3.69~7.19)	8.95 (4.43~35.27)	-1.995	0.046
> 7	13/41 (31.7%)	10/17 (58.8%)	3.693	0.055
PCT (ng/ml)	0.04 (0.031~0.052)	0.057 (0.03~0.19)	-1.939	0.053
> 0.5	20/41 (48.8%)	10/17 (58.8%)	0.485	0.486
D-D (mg/l)	0.28 (0.22~0.46)	2.66 (0.66~5.03)	-5.099	0.000
> 0.55	7/41 (17.1%)	12/17 (70.6%)	15.624	0.000
Biochemical indexes of liver				
ALT (u/l)	21.0 (14.0~36.0)	30.0 (14.75~93.75)	-0.842	0.400
> 40	8/41 (19.5%)	8/17 (47.1%)	-	0.052
AST (u/l)	23.0 (18.5~28.5)	28.0 (19.0~47.75)	-1.311	0.190
> 40	7/41 (17.1%)	5/17 (29.4%)	-	0.307
Alb (g/l)	38.49 ± 4.49	32.28 ± 6.29	1.789	0.186
TBil (μmol/l)	11.0 (7.70~14.75)	11.8 (8.23~14.5)	-0.226	0.821
ALP (u/l)	66.0 (58.5~85.0)	64.5 (46.5~79.25)	-1.118	0.264
GGT (u/l)	23.0 (13.0~42.5)	51.0 (19.25~63.0)	-2.534	0.011
Cardiac function indexes				
LDH (u/l)	199.5 ± 150.09	292.94 ± 113.34	-3.378	0.003
> 245	5/41 (12.2%)	10/17 (58.8%)	-	0.001
hcTnT (ng/ml)	0.004 (0.003~0.006)	0.0065(0.003~0.016)	-1.571	0.116
> 0.1	2/41 (4.8%)	5/17 (29.4%)	-	0.019
CK-MB (ng/ml)	0.65 (0.47~1.01)	1.12 (0.60~2.15)	-2.363	0.018
BNP (pg/ml)	33.15 (12.49~137).	276.35 (59.30~743.88)	-3.900	0.000
> 125	10/41 (24.4%)	10/17 (58.8%)	6.307	0.012

***Note***: COVID-19, Corona Virus Disease 2019; WBC, white blood cell count;

ANC, absolute neutrophil count; ALC, absolute lymphocyte count; CRP, C reactive protein;

IL-6, interleukin-6; PCT, procalcitonin; D-D, D-Dimer; ALT, alanine aminotransferase;

AST, aspartate aminotransferase; Alb, albumin; TBil, total bilirubin; ALP, alkaline phosphatase;

GGT, gamma-glutamyltransferase; LDH, lactic dehydrogenase;

hs-cTnT, high-sensitivity cardiac troponin T; CK-MB, creatine Kinase Isoenzyme-MB;

BNP, brain natriuretic peptide; and -, the Fisher exact probability method was used, without statistic.

There was no significant difference in ALT, AST, Alb, TBil or ALP between the severe group and the mild group (*p* > 0.05). However, there was a significant difference in the GGT between the mild group and the severe group (*p* < 0.05) (Table-II).

For cardiac function indexes in COVID-19 patients, LDH, cTnT, CK-MB and BNP were positively correlated with the severity of the disease, and there were significant differences in LDH, CK-MB and BNP between the mild group and the severe group (*p* < 0.05) (Table-II).

## DISCUSSION

The clinical symptoms of COVID-19 patients mainly included fever, cough, fatigue and dyspnea. At present, the diagnosis of COVID-19 was still based on the positive result of viral nucleic acid detection. It has been reported that there was a high false negative in nucleic acid detection, so the initial chest CT examination became particularly important.^6^ Pan et al. found that patients in the early stage of COVID-19 were mainly presented with GGO, which may be related to the injury of alveolar wall, congestion of blood vessels and edema of alveolar septum caused by COVID-19 infection.^7^,^8^ With the progression of COVID-19, the consolidation of lesions gradually increased. Lung consolidation reflected the high-density shadow formed by the fact that air in the alveolar cavity was replaced by inflammatory cells, edema and bleeding. It was believed that the pleural effusion in patients with severe COVID-19 was caused by pleurisy or reactive inflammation.^9^ The data in our study showed that both lungs were involved with COVID-19 lesions in the severe group, and we found that the percentage of COVID-19 lesions in the volume of the whole lung was positively correlated with the clinical types, suggesting that accurate quantitative assessment indicators could be provided, which was beneficial to evaluate the severity of the lesion and the dynamic changes of patient’s condition.

According to the peripheral blood examination results, normal or decreased WBC, increased neutrophil count (NC), decreased lymphocyte count (LC) and elevated CRP and IL-6 were observed. Moreover, there were significant differences in NC, LC, CRP and IL-6 between the mild group and the severe group, which was consistent with findings reported by Liu Y et al., suggesting that patients infected with COVID-19 may suffer from multiple organ dysfunction.^10^,^11^ Previous studies have shown that increased serum levels of pro-inflammatory cytokines were associated with lung inflammation and lung injury in patients.^12^-^14^ Our study found that the level of IL-6 in the severe group was significantly higher than that in the mild group. IL-6 was a clinically recognized laboratory index for the diagnosis of pulmonary infection.^15^ Lymphocytes were demonstrated to be very important for maintaining immune system function and virus clearance.^16^,^17^ The phenomenon of increased WBC and LC and decreased NC were observed in a small number of patients in the mild group and the severe group, considering that some patients may be associated with other bacterial pneumonia, and the phenomenon may be related to autoimmune mechanism.

The study showed that some COVID-19 patients may be associated with different degrees of liver biochemistry abnormalities, and ALT and AST higher than the upper limit of normal reference value were found in 16 cases (27.6%) and 12 cases (20.7%,), respectively, with the median slightly higher in the severe group than that in the mild group. Liu et al. reported that the proportion of liver biochemical index abnormality in 32 COVID-19 patients was not obviously high, which was consistent with our results.^18^ We also found that GGT was significantly different between the two groups. Chen et al. showed that 43 cases (43.40%) had different degrees of liver dysfunction.^19^ In addition, patients with COVID-19 had varying degrees of hypoxemia, and oxygen therapy was needed in more than 40% of COVID-19 patients suffering from hypoxemia_._^20^ Ischemia and hypoxia may be one of the main mechanisms of liver injury in patients with severe and critical COVID-19. Patients with severe COVID-19 may be more likely to develop liver dysfunction. Therefore, the monitoring and evaluation of liver function during the treatment of patients with severe COVID-19 should be strengthened.

A report by Huang et al. published in Lancet mentioned that, of 41 patients with initially confirmed COVID-19, five cases (12%) had acute myocardial injury, and abnormal electrocardiogram between severe patients and mild patients.^21^ Our data showed that there were certain correlations between the cardiac function indexes and the severity of COVID-19. LDH, CK-MB and BNP were increased with the aggravation of COVID-19, and there were significant differences in LDH, CK-MB and BNP between the mild group and severe group. It has been revealed that increased LDH, CK-MB and BNP may be related to myocardial damage, and the cause of myocardial damage may be related to inflammatory factors or imbalance of oxygen supply.^22^

### Limitations

It includes a relatively small sample size; failure of investigating the relationship between clinical types and the presence of underlying diseases besides a failure to analyze the prognosis of patients Therefore, more in-depth and multi-faceted studies were still needed in the further.

## CONCLUSION

The CT features at initial diagnosis of COVID-19 were mainly characterized by morphology and distribution of multiple GGO in both lungs. There was correlation between some lab test indexes and the clinical types of COVID-19, thus the comprehensive analysis of the imaging findings and the clinical features of COVID-19 patients was helpful for doctors to determine the onset condition and to predict the severity of patient’s condition, with a certain reference value.

### Authors’ Contributions:

**HR & XZ:** Designed this study, prepared this manuscript, are responsible and accountable for the accuracy and integrity of the study. **YL & TY:** Collected and analyzed clinical data. **YT:** Significantly revised this manuscript.
